# Vacancy-controlled ultrastable nanoclusters in nanostructured ferritic alloys

**DOI:** 10.1038/srep10600

**Published:** 2015-05-29

**Authors:** Z. W. Zhang, L. Yao, X.-L. Wang, M. K. Miller

**Affiliations:** 1 Oak Ridge National Laboratory, Oak Ridge, TN 37831, USA; 2 Department of Physics and Materials Science, City University of Hong Kong, Kowloon, Hong Kong

## Abstract

A new class of advanced structural materials, based on the Fe-O-vacancy system, has exceptional resistance to high-temperature creep and excellent tolerance to extremely high-dose radiation. Although these remarkable improvements in properties compared to steels are known to be associated with the Y-Ti-O-enriched nanoclusters, the roles of vacancies in facilitating the nucleation of nanoclusters are a long-standing puzzle, due to the experimental difficulties in characterizing vacancies, particularly in-situ while the nanoclusters are forming. Here we report an experiment study that provides the compelling evidence for the presence of significant concentrations of vacancies in Y-Ti-O-enriched nanoclusters in a nanostructured ferritic alloy using a combination of state-of-the-art atom-probe tomography and in situ small angle neutron scattering. The nucleation of nanoclusters starts from the O-enriched solute clustering with vacancy mediation. The nanoclusters grow with an extremely low growth rate through attraction of vacancies and O:vacancy pairs, leading to the unusual stability of the nanoclusters.

A high density of Y-Ti-O-enriched nanoclusters (NCs) has been recently recognized to play a key role in the design of a promising new class of advanced structural materials with exceptional resistance to high-temperature creep and excellent tolerances to high-dose radiation^1^,^2^,^3^,^4^,^5^. These NCs improve the alloy’s creep resistance by six orders of magnitude between the temperatures 650–900 °C^6^,^7^ and also present an extremely high resistance to coarsening and dissolution under displacement cascade damage in harsh particle irradiation environments^8^.

Y-Ti-O-enriched NCs have been extensively investigated. Atom probe tomography (APT) has revealed that these clusters are extremely fine with diameters of 1–4 nm, and are basically composed of Y, Ti and O, as well as significant amounts of Fe and Cr^2^,^9^,^10^,^11^,^12^. Small angle neutron scattering (SANS) has also revealed that the number densities and volume fractions of the NCs decreased, and their radii increased, with increasing consolidation temperature^12^. The NCs have a defective NaCl structure and a strong structural affinity with the bcc matrix, as determined through a combination of scanning transmission electron microscopy (STEM) observation and theoretical simulation^1^. This defective structure involves large amount of vacancies. The NCs were found to be coherent with the Fe matrix, with truncated rhombic dodecahedron morphologies defined by the {100} and {110} planes^12^. The current knowledge of the NCs in ferritic alloys was mostly obtained from hot-extruded alloys^9^,^13^,^14^,^15^. However, a crucial aspect of the formation and stability of Y-Ti-O enriched NCs is imparted by the mechanical alloying process, which provides the mechanism by which Y_2_O_3_ is forced in solid solution as dissociated Y and O atoms. Mechanical alloying also introduces a large number of vacancies^9^,^16^.

Vacancies-assisted nucleation of precipitates has been intensively investigated in variety of materials, from the nucleation and growth of intermetallic nanoclusters on oxide surfaces in metal catalysts system^17^,^18^,^19^,^20^ to oxides in silicon crystals^21^,^22^,^23^ and Magnetic monopoles in spin ice^24^. The effects of vacancy on the nucleation of nanoclusters are strongly dependent on the vacancy configurations, content, and the affinity between vacancy and solute atoms^22^,^23^. Vacancy defects are also known to have a strong influence on the magnetism in oxygen-deficient pyrochlores (Y_2_Ti_2_O_7_), a spin-ice material^24^.

Vacancy clusters containing four to six vacancies have been found in the mechanical alloyed (MA) and extruded oxide dispersion strengthened (ODS) ferritic alloy by positron-life time spectroscopy^14^. The results from positron annihilation life-time studies in ODS steels with and without nanoclusters indicated that positron annihilations occur at high densities of Y-Ti-O enriched nm-scale features, dislocations or in large caviaties or Ar bublles^25^,^26^. It has been proposed that vacancies can be exploited as a constitutive element in this alloy^11^. As the vacancies are an important factor in the formation and stability of NCs, it is vitally important to characterize the vacancy content within the NCs. Positron-lifetime spectroscopy can prove only the existence of vacancies in the alloy, but not their local distributions. Although the NCs have been extensively characterized in ferritic alloys by APT and SANS^27^, neither of these techniques alone can provide a complete characterization of the distribution of the vacancies. In this letter, a thorough study of the NCs is conducted with a combination of *in situ* SANS and APT. The complementary nature of composition-sensitive APT and size-sensitive SANS, provides compelling evidence of the existence of a large number of vacancies in the NCs as a constitutive element. The vacancy concentration was found to vary with annealing time and tends towards approaching the O concentration in the NCs.

## Results

### Morphology and composition of Nanoclusters

**Figure 1:**
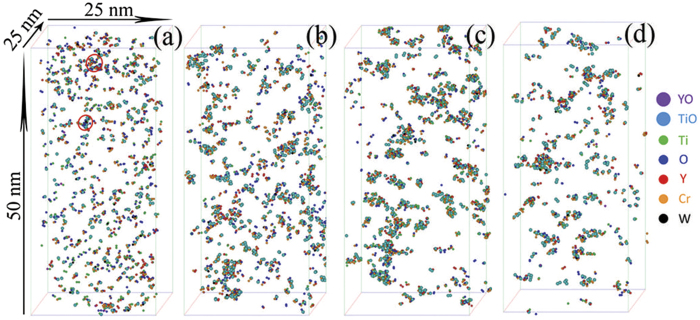
(Zhang). Atom maps revealing the formation of Y-Ti-O enriched nanoclusters in the as-milled specimen (**a**) and the specimens annealed at 500°C for (**b**) 1 h, (**c**) 10 h, and (**d**) 24 h. Solute clustering occur during mechanical alloying and some large solute clusters nucleate, forming the Ti-Y-O nanoclusters (circled in Fig. 1a).

**Figure 2:**
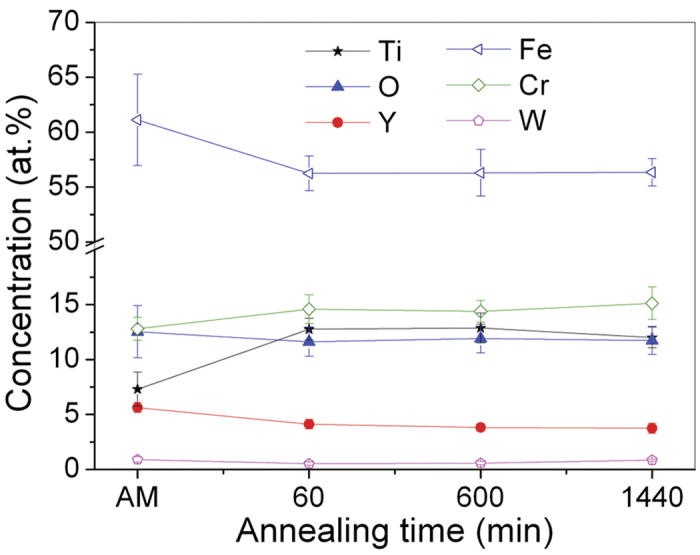
(Zhang) Composition profiles showing the compositions of clusters under various conditions.

Atom maps for the as-milled (MA) and annealed specimens are shown in Fig. 1. High number densities of Ti-Y-O-enriched solute clusters, along with some larger Ti-Y-O-enriched NCs, were identified in the MA specimen (Fig. 1a). This result indicates that either mechanical alloying for 40 h was not sufficient to disperse the solutes randomly, or that some solute clustering occurred during the process. Annealing the MA material for 1 h leads to the formation of large number densities of Y-Ti-O-enriched NCs (Fig. 1b). There was no apparent growth or coarsening of the NCs during annealing for up to 24 h (Fig. 1c,d) demonstrating their ultrahigh stability. The compositions of the clusters following various annealing times are summarized in Fig. 2. In the MA specimen, the solute clusters have significant levels of Fe. After annealing for 1 h, the level of Fe decreases and the concentrations of Y, Ti, and O are ~4, ~12.5 and ~12.5 at.%, respectively. The O/Y ratio is ~3, indicating that mechanical alloying forced the atoms of the Y_2_O_3_ powder into solid solution. This result is consistent with the results observed for hot-extruded specimens^2^,^28^. The Ti/Y and Ti/O ratios are ~3 and ~1, respectively, which indicate that the NCs are not Y_2_Ti_2_O_7_, Y_2_TiO_5_, or TiO_2_ phase^29^, consistent with previous APT results^11^. The concentrations of all the elements in the NCs do not change significantly after annealing between 1 and 24 h; however, the overall concentrations of the Y, Ti, and O in the NCs for the MA material are much lower than those in the extruded specimens, where the O and Ti contents can each approach ~40 at. %^28^,^30^. This result indicates that the formation of Y-Ti-O-enriched NCs for the short aging times at 500 °C within the MA material is still in the early stage.

### Cluster size and volume fraction of nanoclusters

**Figure 3:**
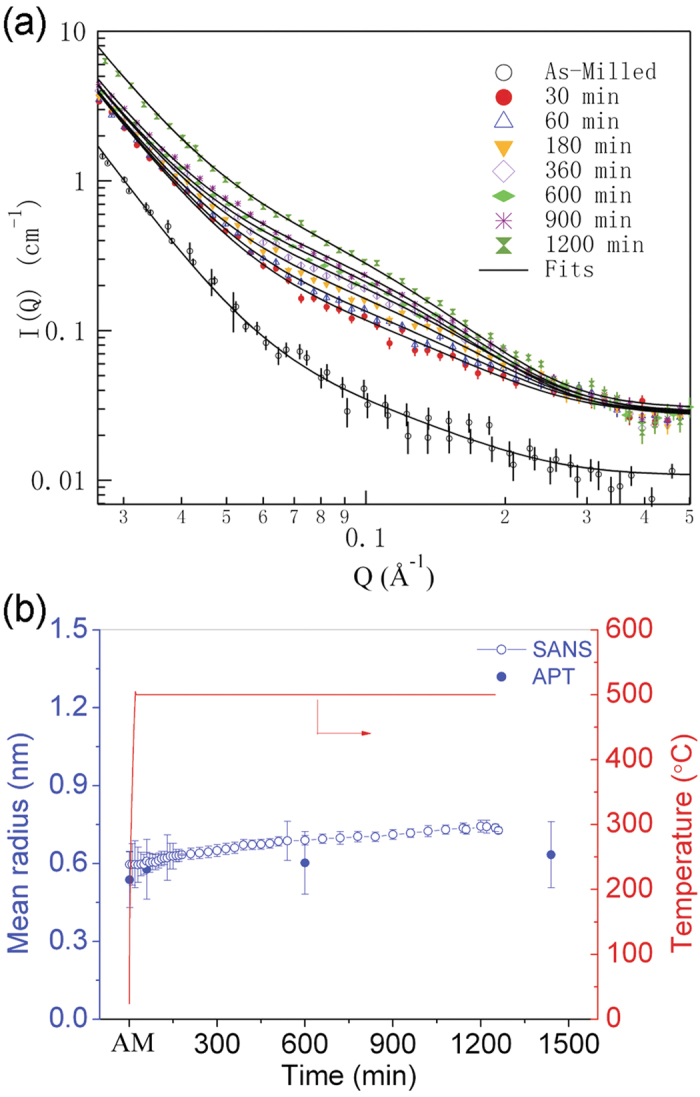
(Zhang). (**a**) Selected SANS-intensity distribution as a function of scattering vector. The solid lines denote the fitting results. (**b**) The nanocluster size determined by *in situ* SANS as a function of annealing time, along with independent estimates from APT data. The temperature profile for *in situ* SANS measurements is also shown in Fig. 3b.

Typical *in situ* SANS data are presented in Fig. 3a. The temperature profile during *in situ* SANS measurement is shown in Fig. 3b. The SANS data can be modeled as the sum of two terms:
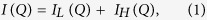
where *I*_*L*_(*Q*) accounts for the scattering at low scattering vector Q, which is dominated by Porod power-law scattering^31^, with
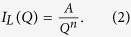
and *I*_*H*_(*Q*) describes the scattering from NCs with a polydisperse log-normal size distribution^32^,
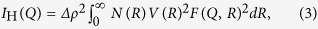
where *R* is the cluster radius, and *V(R)* the cluster volume. *F*(*Q,R*) is the form factor, which can be expressed as *F*(*Q,R*) =3(*sin*(*QR*) −*QRcos*(*QR*))/(*QR*)^3^ for spherical nanoscale clusters. The log-normal size distribution function *N*(*R*) is given by^32^,
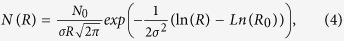
where *N*_0_ is the total number of clusters per unit volume, *R*_0_ is the mean radius, and σ is the polydispersity of the clusters. This polydisperse log-normal size distribution of NCs is verified by the APT results. Δ*ρ* = *ρ*_*m*_ −*ρ*_*c*_ is the difference in scattering length density between the NC *ρ*_*c*_ and the matrix *ρ*_*m*_. With this polydisperse log-normal size distribution of NCs, the mean NC radius is evaluated as <*R*>=exp(*ln*(*R*_0_)+*σ*^2^/2), the mean volume of NCs <*V*>=4*π*/3exp(3*ln*(*R*_0_)+9*σ*^2^/2), and the volume fraction of NCs in the materials ∅ = *N*_0_<*V*>.

Validation of this model was first verified with the wide Q range data obtained before and after *in-situ* annealing. The Porod power-law scattering at low-Q gives an exponent ~5 and ~4 before and after *in-situ* annealing, respectively (see supplementary Fig. S1). This result indicates that the scattering at low-Q arises mainly from grain boundaries^33^. Satisfactory fits to the *in situ* SANS data are obtained with Eq. (1), and are shown in Fig. 2a. The size of the NCs is plotted in Fig. 3b, as a function of annealing time, along with independent size estimates from APT data. The sizes determined from SANS are slightly larger than that determined from the APT data. The APT data shows the NC size increasing slightly after annealing for 1 h as compared to NCs in the MA specimen. This difference arises because of the large number of extremely small solute embryos that co-exist with nucleated NCs and cannot be effectively differentiated. There is no obvious increase in NC size with increasing annealing times up to 24 h; the NC size determined from *in situ* SANS increased by only 0.2 nm. Both APT and SANS data provide clear evidence that the NCs in this alloy are extremely stable during annealing, a trend that is quite different from conventional precipitates, such as Cu-enriched NCs in other ferritic alloys, where the cluster size can increase by a factor of two under similar annealing conditions^27^.

**Figure 4:**
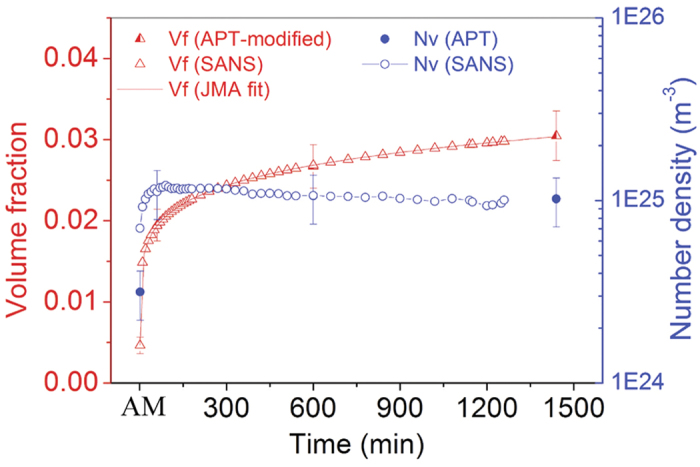
(Zhang). The number density and volume fraction determined from SANS and APT data. The solid line denotes the John-Mehl-Avrami (JMA) fitting results. The fit yielded an exponent n = 0.142 ± 0.004.

With the sizes of the NCs determined from *in situ* SANS, the volume fraction of NCs determined from the APT data was modified because the size in SANS is independent of the composition and therefore, more reliable. The modified volume fraction is shown in Fig. 4. The modified volume fractions were fit using the John-Mehl-Avrami (JMA) transformation theory, which estimates the volume fraction of transformed phase as *x*(*t*)=1−exp(−*k*(*t*−*t*_0_)^*n*^). Satisfactory fits to the modified volume fractions are shown in Fig. 4 (the solid line). The fit yields an exponent *n* = 0.142 ± 0.004, which is often used to characterize the mechanism(s) of phase transformation. The value of *n* decreases to 0.5 when diffusion zones start to overlap (soft impingement) and the transformation rate is retarded^34^, thus, a value of *n* = 0.142 indicates that the NC diffusion zones overlap, which is reasonable in view of the high density of NCs (up to ~10^25^ m^−3^).

### Vacancy concentration in nanoclusters.

**Figure 5:**
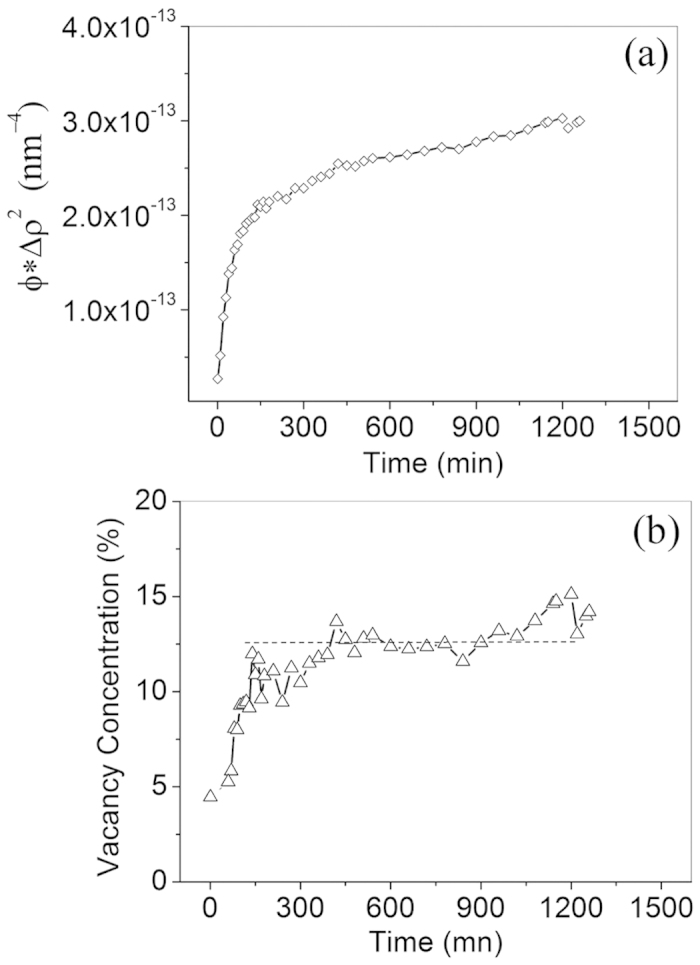
(Zhang). (**a**) Product of volume fraction of clusters, *φ* and the difference in scattering length density of alloy matrix and clusters Δ*ρ*^2^ as a function of annealing time. *φ* and Δ*ρ*^2^ are multiplicative factors and are perfectly correlated. The product of *φ* and Δ*ρ*^2^ is responsible for the scattering intensity and determined by fitting SANS data. (**b**) Vacancy concentration in nanoclusters as a function of annealing time.

*φ* and Δ*ρ*^2^ are multiplicative factors and are perfectly correlated in the model^32^. The product between *φ* and Δ*ρ*^2^, *φ* * Δ*ρ*^2^, can be determined by fitting the SANS data using the proposed model (Eq. (5)). The fitting results of product between *φ* and Δ*ρ*^2^ as a function of annealing time are shown in Fig. 5a. By establishing the NC volume fraction, *φ* as a function of annealing time (Fig. 4), alone with the product from the SANS data fitting (Fig. 5a), the difference in scattering length density Δ*ρ*^2^ can be determined.

The scattering length densities of matrix, *ρ*_*m*_ and clusters, *ρ*_*c*_ can be calculated, as,
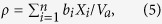
where *b*_*i*_ is the bound coherent scattering length for element *i*, and *X*_*i*_ is the atom fraction of that element in the matrix or NCs. *V*_*a*_ is the average atomic volume. The average atomic volume of elements in matrix is evaluated based on the body-centered cubic (BCC) structure of Fe and the matrix composition determined by APT are used to calculate the scattering length density of matrix. The effect of vacancy on the scattering length densities of matrix *ρ*_*m*_ can be neglected because of the very small amount of vacancies in matrix, as compared to Fe content. With the difference in scattering length density Δ*ρ*^2^ and the scattering length densities of matrix *ρ*_*m*_, the scattering length densities of clusters, *ρ*_*c*_ can be determined.

In this study, the vacancy is defined as an imperfection resulting from an unoccupied element position. To determine vacancy concentration, vacancy in NCs is treated as a constitutive/solute element^11^ and the bound coherent scattering length of vacancy was set to 0. It is well known that atomic volume of elements can be highly sensitive to electronic bonding configuration/oxidation state, as well as mediated by a high-degree of lattice strain^35^. In general, the determination of individual atom volume in crystals is also dependent on the structural information, such as packing density^36^. However, the atomic structures of the nanoclusters are complicated and very difficult to characterize^1^. To copy with this difficulty, two cases with and without vacancy in clusters are proposed (Case 1 and 2 in supplementary materials) to evaluate the effect of vacancies on the fitting results of volume fraction and the average atomic volume in NCs. The vacancy volume is assumed to be the average atomic volume of elements in the NCs. The corresponding vacancy concentrations are determined, as shown in Fig. 5b. These results provide compelling evidence that a significant number of vacancies are associated with the NCs. the vacancy concentration varies with annealing time. In the MA condition, the vacancy concentration is ~5%, far lower than in the steady state condition. After annealing for 2 h, the vacancy concentration tends towards a steady state value of ~12.5%, which is comparable to the O concentration in the NCs.

## Discussion

The vacancy-assisted nucleation and/or aggregation behavior of clusters have been demonstrated to be influenced by both energetics and kinetics^17^. For instance, the vacancy site on rutile TiO_2_ surface are generally believed to serve as nucleation sites that attract metal adatoms diffusing on the surface due to strong binding of vacancy to adatoms. The kinetic effects tend to favor the aggregation of mobile adatoms at nucleation sites with higher adoption energies through fast diffusion channels^17^,^18^. In As- and Sb-doped silicon crystals, it is energetically favorable to form dopant-vacancy-O complexes, which act as precursors for oxide precipitate nucleation under appropriate conditions^21^,^22^,^23^. Although O normally has an extremely low solubility in α-Fe, it is highly supersaturated in the α-Fe matrix of these nanostructure ferritic alloys due to the mechanical alloying process. Annealing accelerates oxygen clustering (Fig. 1). The nucleation of NCs is initiated by solute clustering, specifically O clustering with Ti, Y, and vacancies. This leads to a lower vacancy concentration during the initial nucleation stage (Fig. 5b). First principle calculations of the binding energies between vacancies, oxygen and other solute atoms in ODS 14YWT systems indicates that vacancies have an exceptionally high affinity for O^35^. The exceptionally strong binding between O atoms and vacancies significantly enhances the formation of oxygen: vacancy (O: vac.) pairs, similar to the dopant-vacancy-O complexes in doped silicon crystal systems. Once an O: vac. pair is formed during the annealing step, O atoms and vacancies move together as a unit^37^, which restricts diffusion and contributes to the ultrahigh stability of the NCs^38^. The continuous increase in NC vacancy concentration during the initial stage of nucleation indicates that the O: vac. pairs are prone to migrate to the NCs, leading to the similar contents of O atoms and vacancies in the NCs (Figs. 2,5b). With the formation of O-vac. pairs, Ti and Y solutes are also attracted to NCs to minimize energy, as Ti and Y have higher affinities for O than Fe, and thereby restrict the formation of TiO_2_^11^,^37^. The attraction of NCs to vacancies is also believed to be an important factor in the formation of NCs with the defective structure, and consequently, the unusual stability of the NCs at high temperatures and under intense neutron radiation^1^,^9^,^39^,^40^.

In summary, this proof-of-concept study provides compelling evidence of the existence of vacancies as constitutive elements in NCs formed in the nanostructured ferritic ODS alloy, 14YWT. Vacancies play an indispensable role in the formation of Y-Ti-O-enriched NCs and are responsible for their ultrahigh stability. The nucleation of NCs initiates from O-enriched solute clustering in the ferrite. The NCs attract vacancies and O:vac. pairs during the initial stage of nucleation and proceed to grow during annealing. The NCs have an extremely low growth rate due to the low O mobility. The vacancy content in the clusters can be compared to the O and Ti concentration when the migration of a vacancy is replaced by an O:vac. pair. The combination of APT and *in situ* SANS can be used to evaluate the vacancy concentration in the NCs, and should find broad applications in the study of nucleation in other vacancy-related physical systems. In addition, control of the diffusion via oxygen-vacancy-solute interactions provides a new path for alloy design in ferritic and other systems.

## Methods

The ODS 14YWT ferritic alloy (nominal composition: Fe-14Cr-3W-0.4Ti (wt.%) with 0.25wt.% Y_2_O_3_) was selected for the experimental investigation. The ferritic 14YWT alloy was processed by mechanical alloying for 40 h using attrition milling in an argon atmosphere in a water-cooled vessel. The MA flakes were used to perform the *in situ* SANS and APT measurements. As the NCs are formed during the hot extrusion consolidation process, the MA flakes were studied prior to hot-extrusion, such that the formation of the NCs can be tailored accurately for both temperature and time schemes. The as-milled powders were annealed at 500 °C for 1 h, 10 h and 24 h, to study the different stages of precipitation using APT. A CAMECA Instruments Inc. LEAP™ 4000X HR local electrode atom probe was used to determine the NC composition, morphology and the distribution of solutes^31^. The focused-ion-beam–based APT sample preparation and data analysis methods are described elsewhere^41^. The number of atoms collected from APT was estimated based on the calibrated detection efficiency of the single atom detector. No evidence of ion trajectory crossings were encountered in this ODS material from field ion microscopy images and field evaporation images. The atom number and composition in a nanocluster are determined by APT through an average of hundreds of clusters. To reveal the formation process of the NCs, *in situ* SANS experiments were conducted at the CG-2 beamline at the High Flux Isotope Reactor (HFIR) at Oak Ridge National Laboratory (ORNL). The as-milled powders were compacted at room temperature into 10 mm diameter and 1 mm thick samples for the *in situ* SANS measurement, which were conducted at 500 °C for up to 20 h. High-quality SANS data were collected with a detector-sample distance of 1.5 m and 4.75 Å neutrons. The SANS data were corrected for the density of the compacted powder, transmission, background, empty beam scattering, detector sensitivity, and placed on an absolute scale using the direct beam method. Thus, the scattering intensity from the reduced data directly represents the differential macroscopic scattering cross-section.

## Additional Information

**How to cite this article**: Zhang, Z. W. *et al*. Vacancy-controlled ultrastable nanoclusters in nanostructured ferritic alloys. *Sci. Rep.* **5**, 10600; doi: 10.1038/srep10600 (2015).

## Supplementary Material

Supporting Information
