# Supplementary material for: Vacancy-controlled ultrastable nanoclusters in nanostructured ferritic alloys
Source: Sci Rep. 2015 May 29;5:10600. doi: 10.1038/srep10600 (PMC4650664; doi:10.1038/srep10600)

**Vacancy-controlled ultrastable nanoclusters in nanostructured ferritic alloys**

Z. W. Zhang^1, 2,*^, L. Yao^2^, X.-L. Wang^3^, and M. K. Miller^2^

^1^ Current address Key laboratory of Superlight Materials and Surface technology, Ministry of Education, College of Materials Science and Chemical Engineering, Harbin 150001, China

^2^ Oak Ridge National Laboratory, Oak Ridge, TN 37831, USA

^3^ Department of Physics and Materials Science, City University of Hong Kong, Kowloon, Hong Kong

**Supplementary information**

**1. Validation of the SANS model**

*In situ* SANS can measure only one Q range from ~0.02-0.06Å^-1^. However, Porod power-law scattering mainly dominates the low-Q range, which can influence the information of nanoclusters determined from the scattering in high-Q range. To check the validation of the model proposed in this study, the data obtained before and after *in situ* annealing with wide Q range were analyzed, as shown in **Fig. S1**. The proposed model can be used perfectly to fit the data. The fitting results give an exponent of ~5 and ~4 for preheat and postheat data, respectively. The exponent of ~5 accounts for the fuzzy boundary, whereas ~4 accounts for the smooth boundary. This is reasonable since the mechanical alloying process reduces the grain size down to ~20 nm, distorts the grains and produces a fuzzy boundary. Annealing recovered some of the distortion of grains and made the boundary smoother (Z. W. Zhang, et al., *Mater. Sci. Eng.*, A379 (2004) 148-153). APT results indicate only the segregation of tungsten and chromium across the grain boundary in our previous paper (L. Yao et al., Scripta Mater. 69:622(2013)).

**2. Modification of the volume fraction determined from APT**

Theoretically, both SANS and APT along can provide information on the volume fraction and number density of nanoclusters. However, to determine the volume fraction from SANS, the difference in scattering length density needs to be known because the volume fraction and the difference in scattering length density are multiplicative factors and are correlated in the model. Only the product between
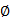
 and
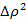
 can be determined by fitting the SANS data. Without the information on vacancy concentration in the nanoclusters, the difference in scattering length density cannot be determined. Therefore, SANS cannot provide information on volume fraction. In order to determine the vacancy concentration of nanoclusters, the volume fraction need to be determined from APT data. APT experiments can independently estimate the volume fraction from
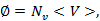
 where
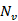
 is the number density and
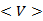
 is the mean volume of clusters. The number density derived from APT measurements is reliable whereas, the radius of clusters could be underestimated, due to differences in the local magnification of the phases and as APT cannot detect vacancies in the nanoclusters. The SANS size is more reliable, as the vacancy are likely to be incorporated in the nanoclusters and the size is independent of the composition. Thus, the volume fraction of nanoclusters in the matrix was modified with the use of the SANS size, as shown in Fig.S2

**3. Comparison of the models with and without vacancies**

In this study, the vacancy concentration in the nanoclusters was determined by treating vacancy as a constitutive element. Two cases are proposed: case 1, treating the vacancy as a constitutive element in the nanoclusters, and case 2, assuming that there were no vacancies in the nanoclusters. To confirm the existence of vacancies in the nanoclusters, the volume fraction and number density of nanoclusters, based on case 2 along with independent estimates from APT data, are presented in Fig. S3 and compared to case 1 (Fig. 3). In case 2, the scattering length densities of matrix and clusters are estimated as
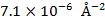
 and
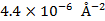
, respectively based on the APT composition. It can be seen from Fig. S3 that both the volume fraction and number density from case 2 are quite different from those from APT, indicating that the case without considering vacancy is not valid. This result further confirms the reliability of case 1, as shown in Fig. 3.

**Supplementary Figure Captions:**

FIG.S1. SANS-intensity distribution as a function of scattering vector for the specimens before (preheat) and after (postheat) *in situ* annealing. The solid lines denote the fitting results.

FIG.S2. Number density and volume fraction of clusters from APT. The half solid triangles demonstrate the volume fraction modified by the cluster radius from SANS.

FIG.S3. Number density and volume fraction of clusters determined based on case 2 (assuming no vacancies in clusters), along with independent estimates from APT data. The half solid triangles demonstrate the volume fraction modified by the cluster radius from SANS. Both density and volume fraction from SANS are quite different from that from APT, indicating that case 2 is not valid.

**Figures**


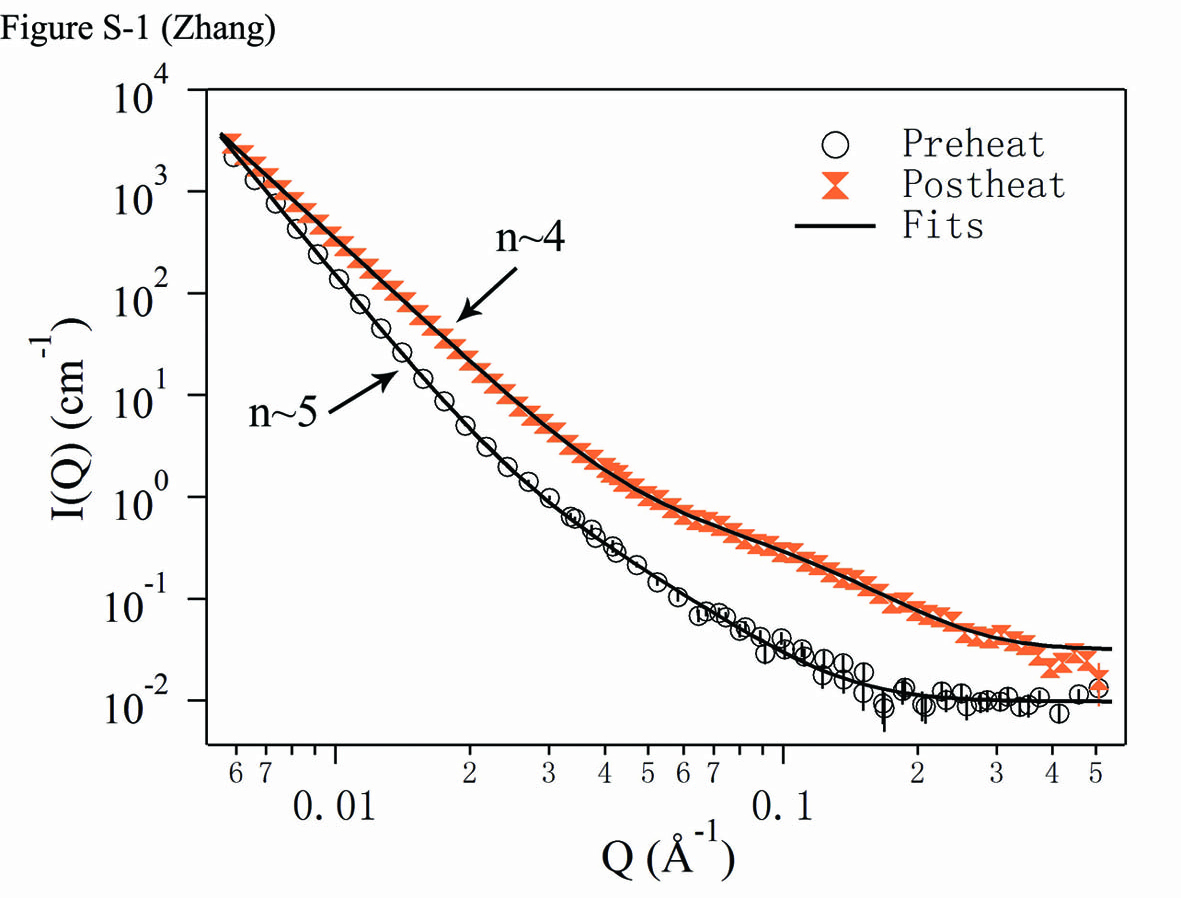


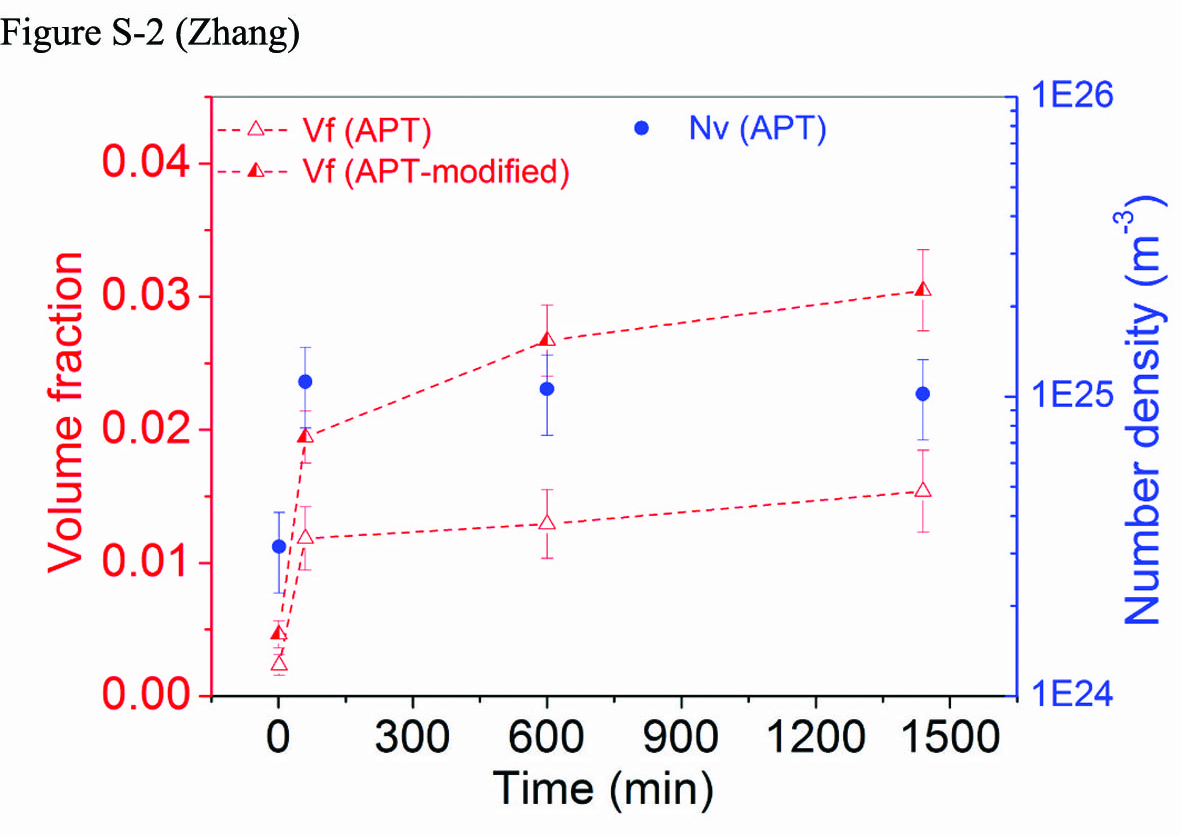


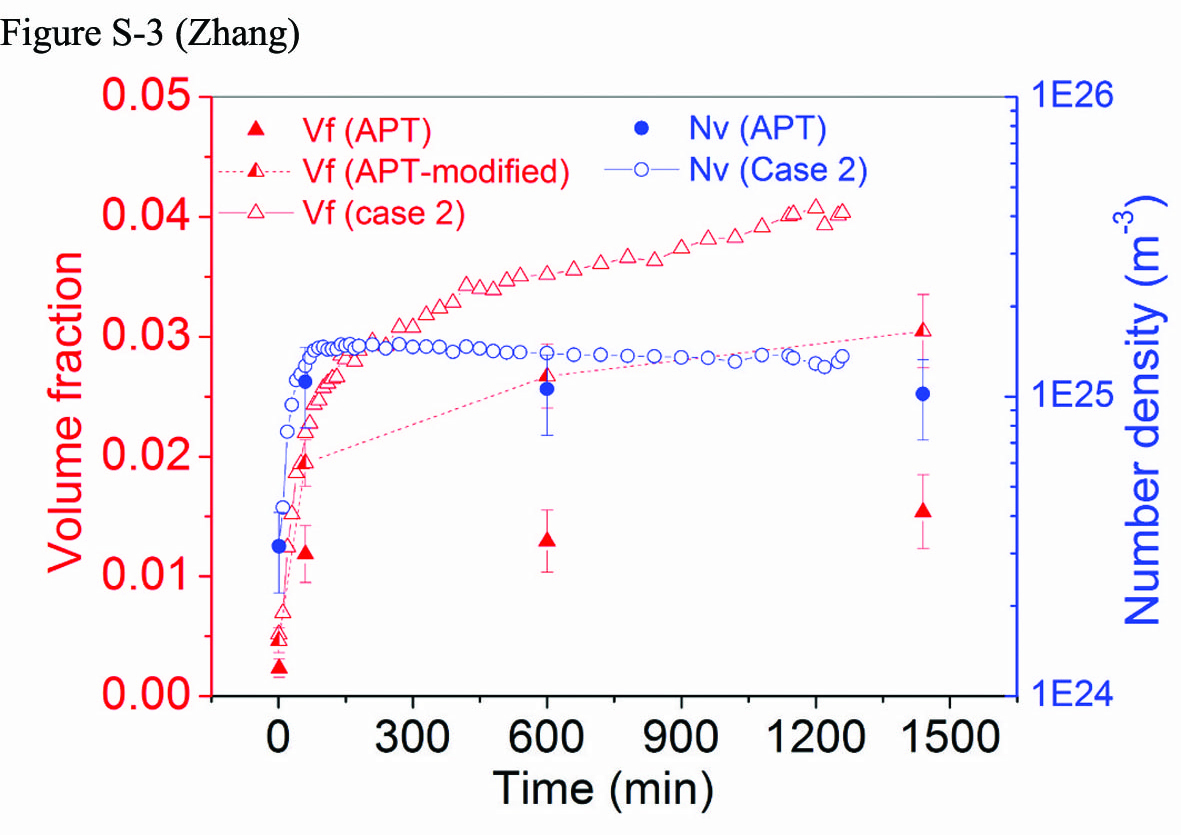

Supplement: Supporting Information [file srep10600-s1.doc]
